# Purinergic Signaling Mediates PTH and Fluid Flow-Induced Osteoblast Proliferation

**DOI:** 10.1155/2021/6674570

**Published:** 2021-01-27

**Authors:** Yanghui Xing, Liang Song, Yingying Zhang, Tengyu Zhang, Jian Li, Chunjing Tao

**Affiliations:** ^1^Department of Biomedical Engineering, Shantou University, Shantou 515063, China; ^2^National Research Center for Rehabilitation Technical Aids, Beijing 100176, China; ^3^Beijing Advanced Innovation Centre for Biomedical Engineering, Beihang University, Beijing 100083, China

## Abstract

Both parathyroid hormone (PTH) and mechanical signals are able to regulate bone growth and regeneration. They also can work synergistically to regulate osteoblast proliferation, but little is known about the mechanisms how PTH and mechanical signals interact with each other during this process. In this study, we investigated responses of MC3T3-E1 osteoblasts to PTH and oscillatory fluid flow. We found that osteoblasts are more sensitive to mechanical signals in the presence of PTH according to ERK1/2 phosphorylation, ATP release, CREB phosphorylation, and cell proliferation. PTH may also reduce the osteoblast refractory period after desensitization due to mechanical signals. We further found that the synergistic responses of osteoblasts to fluid flow or ATP with PTH had similar patterns, suggesting that synergy between fluid flow and PTH may be through the ATP pathway. After we inhibited ATP effects using apyrase in osteoblasts, their synergistic responses to mechanical stimulation and PTH were also inhibited. Additionally, knocking down P2Y2 purinergic receptors can significantly attenuate osteoblast synergistic responses to mechanical stimulation and PTH in terms of ERK1/2 phosphorylation, CREB phosphorylation, and cell proliferation. Thus, our results suggest that PTH enhances mechanosensitivity of osteoblasts via a mechanism involving ATP and P2Y2 purinergic receptors.

## 1. Introduction

Mechanical signals play very important roles in regulating bone growth and remodeling [1, 2]. Oscillatory fluid flow, a potent and widely used mechanical stimulus for bone cells, can induce osteoblast intracellular calcium mobilization, MAPK activation, ATP release, c-fos expression, and other intracellular events and subsequently regulates bone metabolism [3–5]. On the other hand, parathyroid hormone (PTH) is able to regulate bone cell extracellular calcium and phosphate metabolism and controls bone growth and remodeling [6, 7]. It is well established that the intermittent administration of PTH stimulates bone formation by increasing osteoblast number and subsequently increases bone mass and reduces the incidence of fracture in the elderly [8, 9]. PTH enhances osteoblast proliferation, inhibits osteoblast apoptosis, and reactivates lining cells to resume their matrix synthesizing function through a series of pathways, including cAMP, PKA, Runx2, and Wnt signals [9–11]. Both mechanical stimulation and PTH are able to regulate osteoblast metabolism and bone remodeling; thus, their synergistical effects on bone are of great interests of researchers. Previous literatures demonstrated that PTH may potentiate response of bone cells to mechanical stimulation in terms of intracellular calcium release [12, 13]. It is possibly because that PTH induced actin polymerization in cells, which resulted in an increase in cell mechanosensitivity [13]. In addition, PTH has been shown to increase the activity and conductance of a stretch-activated ion channels in osteoblastic cells [14], suggesting its direct action on the cell membranes. Meanwhile, an animal study demonstrated that PTH enhanced mechanically induced bone formation [15]. These studies demonstrated that PTH and mechanical stimulation can work together to improve bone growth and regeneration, but the underlying mechanism remains elusive.

Under mechanical stimulation, osteoblasts release ATP from cytosol to extracellular space. Subsequently, ATP binds to purinergic receptors such as P2Y2 and initiates intracellular calcium mobilization and ERK1/2 activation [4, 16]. ATP is also responsible for fluid flow-induced prostaglandin E2 release in osteoblasts [17]. Furthermore, ATP is able to mediate osteoblast growth and mineralization [18, 19]. ATP is also able to stimulate primary human adipose tissue-derived stem cells and bone marrow stromal cell proliferation through the ERK1/2 pathway and intracellular calcium mobilization, respectively [20, 21]. On the other hand, ATP can modulate bone cell metabolism synergistically with PTH. Osteoblasts may release ATP and become more sensitive after PTH treatment [22]. It has been shown that when ATP and PTH are added together on UMR-106 rat osteoblasts and primary human osteoblasts, there is a synergistic increase in intracellular calcium release and c-fos expression [23, 24]. Thus, ATP is closely associated with the osteoblast mechanotransduction pathway and PTH pathway.

Previously, we have demonstrated that P2Y2 receptor is involved in bone growth and remodeling as well as osteoblast mechanotransduction [4]. Thus, P2Y2 may also have important functions in the synergistic effects resulted from combining mechanical stimulation and PTH. In this study, ERK1/2 phosphorylation was used as a major indictor to measure osteoblast activities because ERK1/2 can initiate many downstream pathways such as COX-2, Runx2, OPG, OPN, and MMP13 and subsequently plays a key role in regulating bone cell activities, including migration, survival, proliferation, and differentiation [25, 26]. We also examined phosphorylated CREB and cell proliferation in order to study mechanisms of the synergy between mechanical stimulation and PTH, which are closely related to bone regeneration.

## 2. Materials and Methods

### 2.1. Cell Culture and Experiment Protocols

The mouse osteoblastic cell line MC3T3-E1 was cultured in minimal essential *α* medium (MEM-*α*) containing 10% fetal bovine serum, 1% penicillin, and streptomycin and maintained in a humidified incubator at 37°C with 5% CO_2_. For oscillatory fluid flow experiments, all cells were cultured on glass slides for 2 days prior to experiments, and 1.5 × 10^5^ cells were seeded on glass slides (75 × 38 × 1.0 mm) for oscillatory fluid flow experiments. Cells were exposed to oscillatory fluid flow in MEM-*α* without FBS for all experiments. The fluid flow chamber employed in this study is a parallel plate design, and the flow delivery device generated 1 Hz sinusoidal oscillating flow with 10 dyne/cm^2^ fluid shear stress on cells as described in previous studies [16, 27]. For ATP effects, 10 mM final concentration ATP was added to cell culture plates directly. For PTH effects, 50 nM final concentration PTH (1-34) was added to cell culture plates or fluid flow medium directly.

### 2.2. Overexpression and siRNA Knockdown of P2Y2

To overexpress P2Y2 in cells, full length cDNA plasmid pcDNA3-P2Y2 was transfected into MC3T3-E1 osteoblasts using the FuGENE 6 transfection reagent kit from Roche according to manufacturer's protocols. For siRNAs against P2Y2, si-P2Y2 sense/antisense was designed and manufactured by Qiagen, Inc. The efficiency of knocking down was confirmed by mRNA and protein expression using RT-PCR and western blotting, respectively.

### 2.3. Western Blot

Immediately after experiments, cells were lysed with RIPA buffer, and total protein concentrations were measured by the bicinchoninic acid assay. Twenty-five micrograms of the total protein from each sample was separated by SDS–PAGE and transferred to PVDF membranes. Membranes were incubated with the desired primary antibody overnight at 4°C and subsequently with the secondary antibody for 1 hour. Visualization of proteins was achieved by using an ECL detection system and membrane exposure to film.

### 2.4. ATP Detection

Samples of conditioned media were collected, and ATP concentration in the samples was measured using a Roche ATP bioluminescence kit. Briefly, ATP was used to convert D-luciferin into oxyluciferin and light. The light density from each sample was measured by a luminometer and compared with a standard curve created by ATP standards. Three measurements were taken from each sample. Results were normalized to protein concentration using the bicinchoninic acid assay.

### 2.5. Cell Proliferation Assay

Cell proliferation was measured with the FITC 5-bromo-2-deoxyuridine (BrdU) flow kit according to the manufacturer's protocols. After exposed to oscillatory fluid flow for 1 hour, MC3T3-E1 osteoblasts were placed back into incubator for 24 hours. After that, cells were labeled with BrdU and then quantified using fluorescence-activated cell sorting with a FITC-conjugated antibody specific for BrdU.

### 2.6. Data Analysis

Data are expressed as mean ± standard error (SE). To compare two groups, two-sample Student's *t*-test was used in which sample variance was not assumed to be equal. To compare observations from more than two groups, a one-way analysis of variance was employed followed by a Bonferroni selected-pairs multiple comparisons test. *p* < 0.05 was considered statistically significant.

## 3. Results

### 3.1. PTH Enhances Osteoblast Responses to Mechanical Stimulation

**Figure 1:**
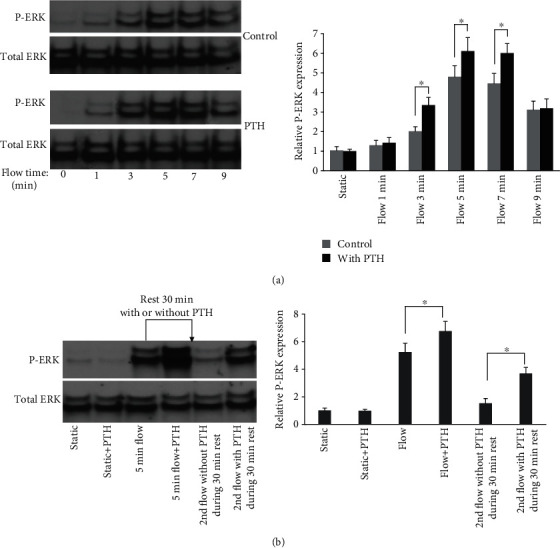
Osteoblast ERK1/2 phosphorylation in response to PTH and oscillatory fluid flow. (a) ERK1/2 phosphorylation in response to fluid flow was significantly increased after PTH treatment. (b) PTH reduces osteoblast desensitization time after mechanical stimulation. (*n* = 4, ^∗^*p* < 0.05, each bar represents the mean ± SEM).

We first examined the effect of PTH on MC3T3-E1 osteoblastic cells in terms of ERK1/2 phosphorylation in response to oscillatory fluid flow. We treated cells with 50 nM PTH for 30 minutes and found that PTH alone did not alter ERK1/2 phosphorylation. However, PTH-treated MC3T3-E1 cells have a significantly greater phosphorylated ERK1/2 expression in response to fluid flow, compared with cells without PTH treatments (Figure 1(a)). Additionally, ERK1/2 phosphorylation quickly reaches its maximum value at round minute 5, then decreases. Thus, we used only 5-minute oscillatory fluid flow in our rest experiments to study ERK1/2 phosphorylation.

### 3.2. PTH Reduces Osteoblast Desensitization Time after Mechanical Stimulation

After subjected to oscillatory fluid flow for 5 minutes, MC3T3-E1 osteoblast may take up to 120 minutes to completely recover its sensitivity to respond to another oscillatory fluid flow according to our previous experiments [28]. In this study, MC3T3-E1 cells were treated with or without PTH for 30 minutes after first fluid flow stimulation, then exposed to second fluid flow stimulation. We found that PTH-treated cells have significantly higher phosphorylated ERK1/2 expression level compared to non PTH-treated cells (Figure 1(b)). The result suggests that PTH may be able to reduce the osteoblast refractory period after mechanical stimulation.

### 3.3. PTH Alters ATP Release Pattern Induced by Mechanical Stimulation

**Figure 2:**
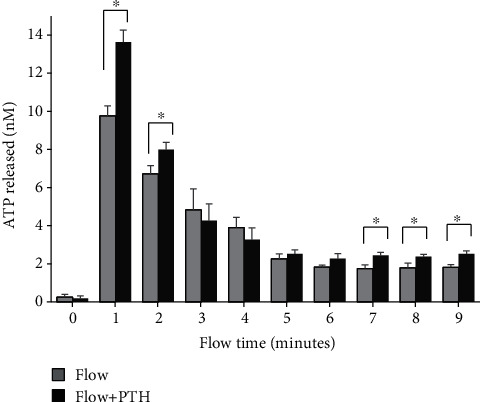
Osteoblast ATP release in response to oscillatory fluid flow with or without PTH. (*n* = 3, ^∗^*p* < 0.05, each bar represents the mean ± SEM).

We also checked ATP release of PTH-treated MC3T3-E1 cells in response to oscillatory fluid flow. We found that the amount of ATP released of PTH-treated cells is significantly increased after 1 minute which is the maximum point of the ATP release curve, compared with nontreated cells. After that, ATP concentration is quickly diminishing. After minute 5, ATP release from cells reached a relatively stable stage, and the changes from minute 5 to minute 9 are very small and insignificant (Figure 2). Similar to the P-ERK expression, the ATP release result suggests that MC3T3-E1 cells are more sensitive to oscillatory fluid flow after PTH treatment.

### 3.4. PTH Enhances Osteoblast Responses to ATP

**Figure 3:**
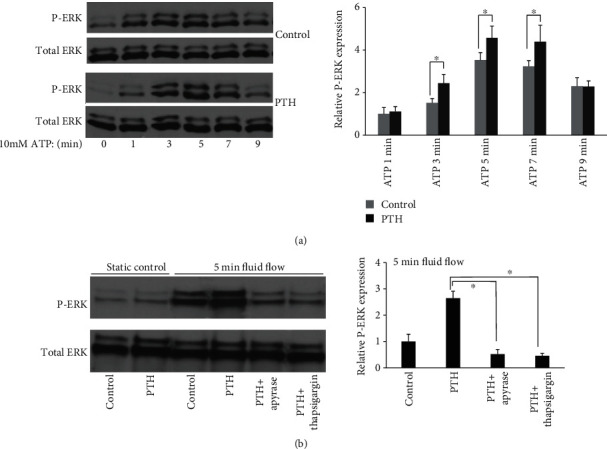
ATP is involved in the synergistic responses of osteoblasts to PTH and oscillatory fluid flow. (a) ERK1/2 phosphorylation in response to ATP with or without PTH treatment for different times. (b) Apyrase and thapsigargin inhibit the synergy between PTH and fluid flow. (*n* = 4, ^∗^*p* < 0.05, each bar represents the mean ± SEM).

Since MC3T3-E1 cells release ATP when subjected to fluid flow stimulation, we also examined the responses of osteoblast after treated with both ATP and PTH. We found that ATP induced ERK1/2 activation, and PTH enhanced this response in a similar pattern to mechanical stimulation. (Figure 3(a)). The results suggest that PTH may potentiate osteoblast responses to mechanical stimulation through the ATP pathway.

### 3.5. The ATP Pathway Is Involved in the Synergistic Responses of Osteoblasts to PTH and Mechanical Stimulation

When we used apyrase (1 U/ml), an enzyme able to rapidly hydrolyzes ATP, together with PTH for MC3T3-E1 during fluid flow experiments, we found that the cell response in terms of ERK1/2 phosphorylation was significantly decreased. If we pretreated cells with thapsigargin (5 *μ*M), an endoplasmic reticulum (ER) ATPase inhibitor, we also found that cell responses to PTH and fluid flow were significantly decreased in terms of ERK1/2 phosphorylation (Figure 3(b)).

### 3.6. PTH May Potentiate Fluid Flow-Induced Effects through P2Y2 Purinergic Receptors

**Figure 4:**
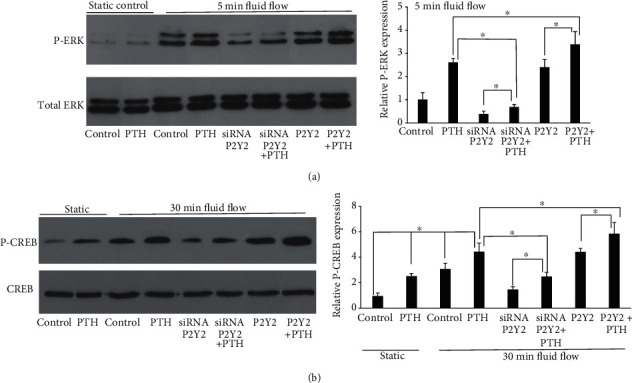
P2Y2 purinergic receptors are involved in PTH and oscillatory fluid flow-induced osteoblast responses. (a) Knockdown of P2Y2 inhibits ERK1/2 phosphorylation while the overexpression of P2Y2 enhances ERK1/2 phosphorylation. (b) Knockdown of P2Y2 inhibits CREB phosphorylation while the overexpression of P2Y2 enhances CREB phosphorylation. (*n* = 4, ^∗^*p* < 0.05, each bar represents the mean ± SEM).

We have showed previously that the P2Y2 receptor is responsible for fluid flow-induced intracellular calcium release and ERK1/2 phosphorylation. In this study, we found that when the P2Y2 expression was knocked down by siRNA in MC3T3-E1 cells, the ERK1/2 phosphorylation level was also significantly decreased even in the presence of PTH. When P2Y2 is overexpressed in MC3T3-E1 cells, the phosphorylated ERK1/2 level was significantly increased with PTH (Figure 4(a)). We also examined CREB phosphorylation when MC3T3-E1 cells were exposed to both PTH and oscillatory fluid flow (Figure 4(b)). We found similar results to ERK1/2 phosphorylation. The results suggest that P2Y2 purinergic receptors may be involved in the crosstalk between the PTH pathway and mechanotransduction pathway of osteoblasts.

### 3.7. ATP and Its Receptor P2Y2 Are Important for Osteoblast Proliferation

**Figure 5:**
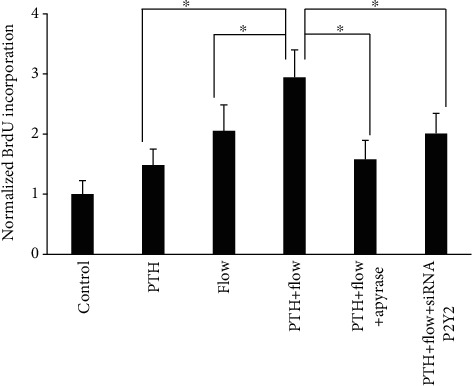
ATP and P2Y2 purinergic receptors are involved in PTH and fluid flow-induced osteoblast proliferation. Removal of ATP by apyrase or knockdown of P2Y2 receptors inhibited PTH and fluid flow-induced cell proliferation. (*n* = 3, ^∗^*p* < 0.05, each bar represents the mean ± SEM).

Osteoblast proliferation is a crucial factor for bone growth and remodeling. So, we examined MC3T3-E1 cell proliferation when exposed to fluid flow and PTH. We found that fluid flow and PTH are able to increase cell proliferation significantly as shown in Figure 5. When combining them together, PTH and fluid flow can increase cell proliferation even greater. When we use apyrase to quickly remove ATP, or use siRNA to knock down the cell P2Y2 expression level, we found that cell proliferation was significantly inhibited.

## 4. Discussion

Mechanical signals mediate bone growth and regeneration, and the intermittent administration of PTH can enhance the effects [15, 29], but the mechanism how they work together is still elusive. We previously examined the role of P2Y2 purinergic receptors in the bone mechanotransduction pathway [4, 30] and found it is closely related to ATP and calcium signaling pathways. Other researchers demonstrated that osteoblast calcium signaling induced by mechanical loads may be enhanced by PTH, and ATP may sensitize PTH receptor [13, 22]. Thus, we believe that P2Y2 purinergic receptors may play an important role in synergistical responses of the combined action of PTH and mechanical signals.

In the present study, we firstly showed that PTH is able to work synergically with oscillatory fluid flow to potentiate osteoblast ERK1/2 phosphorylation, which play important roles in bone formation. The result is similar to previous findings that PTH can enhance the COX-2 expression, calcium release, and c-fos expression from osteoblasts in response to mechanical signals [12, 22, 24]. We also found that PTH is able to reduce the osteoblast refractory period after mechanical stimulation in terms of ERK1/2 phosphorylation. This effect may cause osteoblast response to mechanical signals more often. As a result, osteoblast's total effective response time is increased during mechanical stimulation, which may subsequently lead to further cell proliferation and bone regeneration.

We further found that the osteoblast ATP release induced by oscillatory fluid flow is also enhanced. Since ATP stimulation is able to cause ERK1/2 activation, thus we subsequently examined the synergistic effects of PTH and ATP using osteoblast ERK1/2 activation. We found that PTH is also able to potentiate ATP-induced ERK1/2 activation in the exact same pattern as induced by oscillatory fluid flow. Previous literatures have demonstrated that ATP can synergize with PTH to increase intracellular calcium release and c-fos expression [31, 32]. ATP is also able to synergize with PDGF to increase DNA synthesis in MC3T3-E1 cells [33]. Thus, we suspect that the combined effects of PTH and mechanical signals on osteoblast are through the ATP pathway. For these reasons, we used apyrase to remove fluid flow induced-ATP from extracellular space and checked osteoblast responses. We found that the synergistic effects from fluid flow and PTH were significantly attenuated. Additionally, when we used thapsigargin to inhibit ER calcium mobilization, the synergistic effects were also significantly attenuated. The results suggest that synergy of mechanical signals with PTH may be through the ATP-calcium-ERK1/2 pathway.

Due to the importance of ATP, we examined the role of the P2Y2 purinergic receptor in this process. We knocked down or overexpressed the P2Y2 expression level in MC3T3-E1 cells and compared their synergistic responses to oscillatory fluid flow and PTH to normal MC3T3-E1 cells. We found that P2Y2 knockdown inhibited such synergistic responses in terms of ERK1/2 and CREB phosphorylation. Furthermore, the P2Y2 overexpression in cells enhanced their responses to PTH and mechanical stimulation. These results confirmed that purinergic signaling is involved in the synergy of mechanical signals with PTH. Since both PTH receptor and P2Y2 receptor are GPCRs, they can be desensitized by GPCR kinases (GRKs). We have previous demonstrated that GRKs may be involved in fluid flow-induced osteoblast desensitization [28]. If PTH receptors are desensitized by the same GRK as P2Y2 receptors, then the administration of PTH may lead to recruitment of GRK to PTH receptors. In this situation, desensitization of P2Y2 receptors will be more difficult because the amount of free GRKs is reduced. As a result, PTH can work synergistically with oscillatory fluid flow to enhance osteoblast responses.

Osteoblast proliferation is important for bone growth and regeneration. ERK1/2 has been shown to regulate osteoblast proliferation through a number of pathways, including c-fos and Runx2 [34–37]. For these reasons, we examined the involvement of P2Y2 receptors and ATP in osteoblast proliferation induced by PTH and fluid flow. We found that removal of ATP by apyrase or knockdown of P2Y2 receptors by siRNA inhibited osteoblast proliferation. The results are reasonable since reduction of ATP and P2Y2 inhibited ERK1/2 and CREB phosphorylation based on our previous findings. Additionally, other researchers have demonstrated that purinergic receptors were closely related to cell proliferation [38].

## 5. Conclusions

In this study, we demonstrated that PTH enhanced oscillatory fluid flow-induced osteoblast ERK1/2 phosphorylation, ATP release, CREB phosphorylation, and cell proliferation and decreased the osteoblast refractory period due to mechanical stimulation. We also showed that depletion of extracellular ATP during fluid flow or knockdown of purinergic receptor P2Y2 expression can inhibit above synergistic responses. Our results suggest that osteoblast is more responsive to mechanical signals in the presence of PTH, and P2Y2 purinergic receptors play important roles in the process. Both P2Y2 receptors and PTH receptors are GPCRs, so they may use the same desensitization pathway. As a result, it may become harder to deactivate both of them at the same time.

## Data Availability

The figures and data used to support the findings of this study are included within the article.
